# Real-Time Internet of Medical Things System for Detecting Blood Leakage during Hemodialysis Using a Novel Multiple Concentric Ring Sensor

**DOI:** 10.3390/s22051988

**Published:** 2022-03-03

**Authors:** Hsiang-Wei Hu, Chih-Hao Liu, Yi-Chun Du, Kuan-Yu Chen, Hsuan-Ming Lin, Chou-Ching Lin

**Affiliations:** 1 Department of Biomedical Engineering, National Cheng Kung University, Tainan 704, Taiwan; p88051012@mail.ncku.edu.tw (H.-W.H.); terrydu@gs.ncku.edu.tw (Y.-C.D.); 2 International Academia of Biomedical Innovation Technology, Taipei 104, Taiwan; ky.kychen@acusense.com.tw; 3 AcuSense BioMedical Technology Corp., Tainan 744, Taiwan; alvinliu66@gmail.com; 4 Medical Device Innovation Center, National Cheng Kung University, Tainan 701, Taiwan; 5 Department of Nephrology, An Nan Hospital, China Medical University, Tainan 709, Taiwan; d12598@mail.tmanh.org.tw; 6 Department of Neurology, National Cheng Kung University Hospital, College of Medicine, National Cheng Kung University, Tainan 704, Taiwan

**Keywords:** hemodialysis, venous needle dislodgement, blood leakage detection, multi-bed monitoring system, Internet of Medical Things (IoMT)

## Abstract

Venous needle dislodgement (VND) is a major healthcare safety concern in patients undergoing hemodialysis. Although VND is uncommon, it can be life-threatening. The main objective of this study was to implement a real-time multi-bed monitoring system for VND by combining a novel leakage-detection device and IoMT (Internet of Medical Things) technology. The core of the system, the Acusense IoMT platform, consisted of a novel leakage-detection patch comprised of multiple concentric rings to detect blood leakage and quantify the leaked volume. The performance of the leakage-detection system was evaluated on a prosthetic arm and clinical study. Patients with a high risk of blood leakage were recruited as candidates. The system was set up in a hospital, and the subjects were monitored for 2 months. During the pre-clinical simulation experiment, the system could detect blood leakage volumes from 0.3 to 0.9 mL. During the test of the IoMT system, the overall success rate of tests was 100%, with no lost data packets. A total of 701 dialysis sessions were analyzed, and the accuracy and sensitivity were 99.7% and 90.9%, respectively. Evaluation questionnaires showed that the use of the system after training changed attitudes and reduced worry of the nursing staff. Our results show the feasibility of using a novel detector combined with an IoMT system to automatically monitor multi-bed blood leakage. The innovative concentric-circle design could more precisely control the warning blood-leakage threshold in any direction to achieve clinical cost-effectiveness. The system reduced the load on medical staff and improved patient safety. In the future, it could also be applied to home hemodialysis for telemedicine during the era of COVID-19.

## 1. Introduction

In 2019, the World Health Assembly (WHA) declared that patient safety is a critical challenge for public health worldwide. As many as 134 million adverse events occur each year in the hospitals of low and middle-income countries, resulting in 2.6 million deaths [1]. The Global Burden of Disease Study estimated that 1.2 million people died of kidney failure in 2015 [2]. Fresenius, a leading dialysis-equipment manufacturer, reported that the number of patients under dialysis was 430 million worldwide in 2016, and that the number is expected to grow by 5–6% each year [3]. Hemodialysis presents a significant safety issue due to fluid leakage and venous needle dislodgement (VND). Minor and critical blood leakage incurs 818 million dollars in terms of medical cost each year in the United States [4]. In 2012, the American Nephrology Nurses’ Association set up a team to examine the occurrence, consequences, and practices related to VND in an attempt to help kidney disease paramedics, patients, and families formulate countermeasures. According to their survey, (1) more than 70% of respondents said that VND was common in those undergoing chronic hemodialysis, (2) 76.6% of respondents said that they had experienced VND in the past 5 years, and (3) 85.3% of respondents said they were willing to receive training programs in order to minimize the occurrence [5]. The risk factors associated with VND include muscle cramp, dementia, incomplete awakening, allergy to adhesive tape, hemodialysis at home without assistance, and overnight hemodialysis [6].

According to the American National Standards Institute, none of the currently available dialysis machines are equipped with reliable mechanisms to monitor critical blood leakage [7]. As a result, the institute has issued the IEC/PAS 63023:2016 (https://reurl.cc/kL4lmb, accessed on 19 November 2021) decree that requires all dialysis machines to be equipped with an input interface that stops the dialysis machine and sounds an external warning in the event of tube slippage or fluid leakage. The currently available commercial products use infrared light to detect fluid leakage [8]. The working principle is a single-point needle detector that differentiates between blood and colorless liquids according to the reflection of infrared light. In clinical applications, the fluid leakage has to exceed 1 mL before being detected [9].

In 2019, Du et al. [10] proposed a portable light-weight monitoring and warning device which consisted of a sensor patch and a warning device to detect blood leakage resulting from needle dislodgement in an arteriovenous fistula (AV fistula), AV graft, and central venous catheter (CVC). The sensor patch was incorporated into a flexible support board and can detect fluid leakage in the range of 0.5 to 1 mL without false warnings from micro-leaks. Their cross-loop circuit sensing is based on multi-channel mapping. This device was tested at a medical center during 544 sessions of hemodialysis, and the results showed that the precision and accuracy of detection were 98.7% and 98.9%, respectively. However, the device failed to detect blood leakage in six clinical trials because the distance between the wrap-around patch and needle insertion point was too small, and the small amount of leaked blood was instantly absorbed by the gauze and did not trigger the warning device [11].

In this paper, we propose a novel system combining a new sensor patch and IoMT (Internet of Medical Things) technology, using BLE (Bluetooth Low Energy) and Wi-Fi. We first completed simulation tests and clinical trials to evaluate the performance of leakage detection. We then used digital technology to develop a real-time multi-bed monitoring system to detect fluid leakage. In addition, the device recorded abnormal events, and provided objective data for clinical review and interpretation. The system facilitated the standard procedure of hemodialysis in tandem with a smart wearable device for clinical use, which facilitates its use by paramedics.

## 2. Material and Methods

### 2.1. Sensor Patch Development

**Figure 1:**
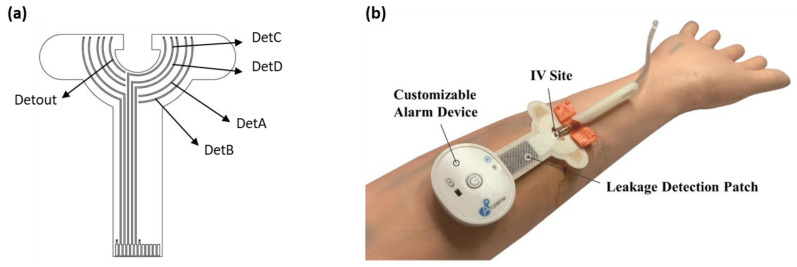
Sensor patch layout and the in vitro setup for testing the device. (**a**) Sensor patch layout. The cross-ring design architecture from the inside out was designed to detect leakage from all directions. (**b**) The performance of the leakage-detection patch was tested on a prosthetic arm with other parts of the detection device.

The proposed device consisted of a multi-ring-shaped sensor (AcuSense BioMedical Technology Co. Ltd., Tainan, Taipei) which started to detect leakages from the inner layer to the outer layer as the amount of liquid increased (Figure 1a). The device (AcuSense BioMedical Technology Co. Ltd., Tainan, Taipei) also included a mapping circuit and a Bluetooth low energy module. Each sensor patch was manufactured using a flexible board-manufacturing process. A signal line was embedded in the insulating layers during production, and only the array sensing point was exposed to the outside. The signal line was also insulated to avoid signal transmission interference. The gap between the sensing lines was 0.5 mm.

The performance of the leakage-detection patch was first tested on a prosthetic arm. The setup, including the positions of the syringe, sensor patch, and leakage device, is shown in Figure 1b. A syringe with a scale of 0.1 mL was used to inject a small amount of pig blood into the leakage test point with a flow rate of 1 mL/s. This experiment was conducted with 0.1, 0.2, 0.3, 0.4, 0.5, 0.6, 0.7, 0.8, 0.9 and 1.5 mL of blood. The tests were run 10 times for each volume. The process from blood leakage to detection consisted of the following three stages: (1) absorption—blood was absorbed by the gauze and diffused to the patch; (2) reaction—blood diffused into the patch causing the detection voltage to rise; and (3) saturation—blood no longer spread and the voltage remained stable. In addition to the detection rate, the delay time from the absorption phase to the saturation phase was also recorded.

**Figure 2:**
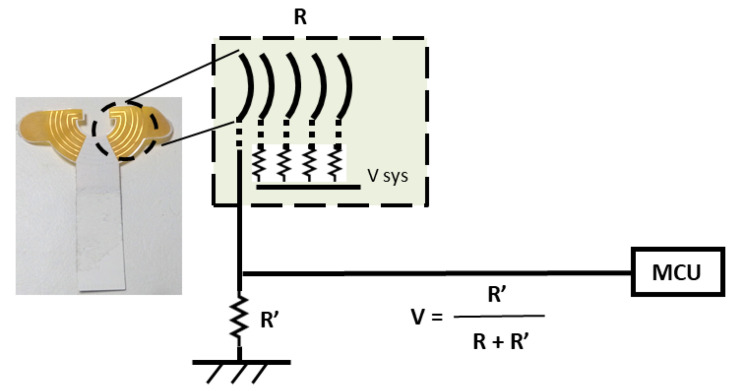
Circuit design of the sensor patch and the principle of cross-ring threshold.

Regarding the cross-loop design of the sensing circuit, the design was based on the principle of an open circuit and parallel resistance (Figure 2). When the liquid passed through the first conductive ring and the second conductive ring, a closed circuit was formed, and the voltage (V) could be measured. The greater the liquid volume, the larger the diffusion area, and more conductive loops formed parallel resistances. The smaller the equivalent resistance (R), the greater the measured V so that the volume of blood leakage could be quantified.

### 2.2. Hardware and Software Development

**Figure 3:**
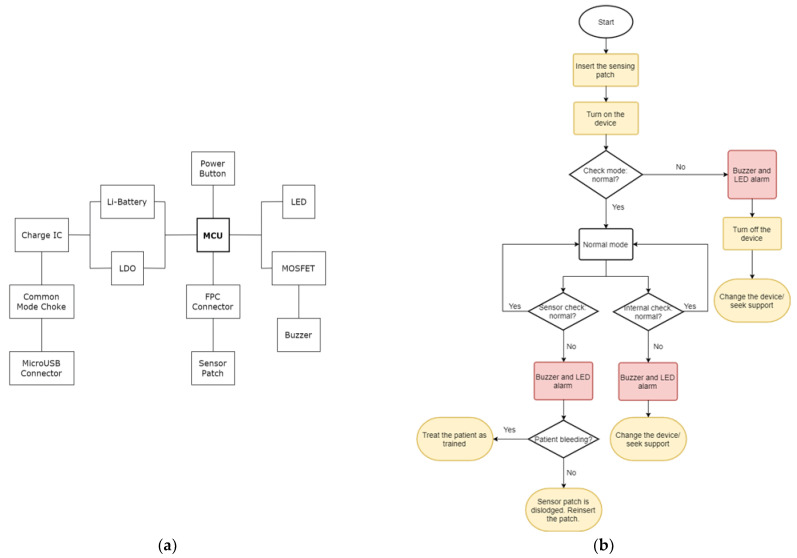
(**a**) Block diagram of hardware circuit design, featuring low power consumption and miniaturized wearable style. (**b**) Flow chart of software design. One-button start-up sensing mode was adopted to make it more convenient and time-saving for medical staff.

The hardware architecture diagram is shown in Figure 3a. A 4.2 V Li-battery was adopted as the battery supply and an LDO (Low Dropout Regulator) module was used to maintain the system voltage at 3.3 V. A disposable FPC (Flexible Printed Circuit) soft board was used to implement the sensor patch, and the FPC connector adopting the wearable host design could be plugged and unplugged many times for multiple use. A set of LEDs was used for warning. When the battery charge was abnormal or the liquid leakage was detected, the MCU (microcontroller) would change the voltage to turn the LED light from green to red. The warning from the buzzer was driven by a MOSFET (Metal-Oxide-Semiconductor Field-Effect Transistor) module, which emitted an intermittent warning sound, and the MCU sent a Bluetooth packet signal every second. In terms of charging management, a microUSB connector was used to connect the power cable, and interference signals were filtered out through a common mode choke, which measured the charging current and the battery voltage with a charge IC to control the charging current and meet the needs of different stages of battery charging. The device could be fully charged in about 1 h.

The software functionality was designed with an emphasis on clinical convenience. Directions for use are as follows: when initiating use, insert the sensor patch, turn on the power supply, press the switch button for one second, and the device will check the potential status of the LED and the buzzer first by itself. A warning signal indicating a malfunction in the device allows the user to replace a backup device in the first instance, and then to carry out inspection and maintenance of the original device. If the device is in a normal state, it automatically enters a continuous blood-leakage detection stage, and also checks the potential status of the LED light and Buzzer continuously. If abnormal, it reports for maintenance. If a warning signal occurs in the sensing circuit, it indicates a possible blood-leakage event. The ADC (analog-to-digital converter) determines whether the change of the sense potential exceeds the preset threshold. If the threshold is exceeded, a liquid-leakage warning is issued, so that the medical staff are able to immediately deal with the needle and re-insert the needle to the normal state. The functional design process is shown in Figure 3b, and its one-button start-up operation mode achieves practical usability for medical staff.

### 2.3. Arm Simulation Experiment

**Figure 4:**
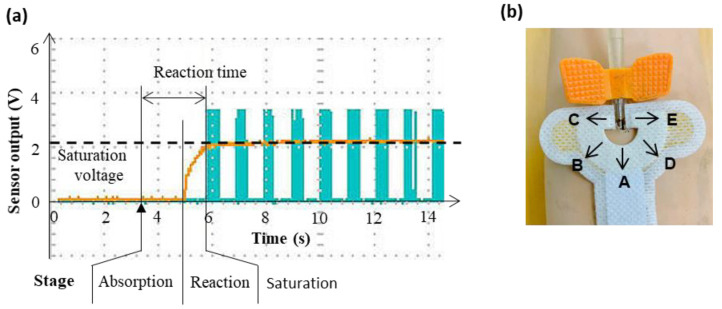
(**a**) Temporal profile of the voltage of the detection patch after injecting a single bolus of pig blood. Yellow line: the voltage from the detection patch. Cyan clusters of spikes: warning buzzers. Red dotted line: the saturation voltage. (**b**) The directions of blood ejection. A–E in (**b**) are 5 directions.

An example of the results from the prosthetic arm simulation experiment to evaluate the performance of the leakage detection patch is shown in Figure 4a. At the injection time, a bolus of 0.5 mL of pig blood was injected at the needle point. The whole temporal profile can be divided into the following three stages: the absorption stage, reaction stage and saturation stage. In the absorption stage, the voltage response remained unchanged as the blood was absorbed by the absorbent pad. About 1 s later, the voltage began to rise (the reaction stage), and finally the voltage reached a steady level (a saturation voltage of 2.155 V) in the saturation stage. The reaction time, defined as the interval from the injection time to the start of buzzing, was 2.46 s.

The complete leakage-detection device consisted of the leakage-detection patch, an alarm unit, a processing unit, and a Bluetooth module. When blood leakage occurs, the processing unit of the leakage detection device receives data from the leakage detection patch, and transmits the processed information via the Bluetooth module to an IoMT gateway.

Two sets of simulation were conducted to evaluate the performance of the sensor. In the first set, the blood was released in five directions (Figure 4b) to test whether the sensor was equally sensitive to all directions. The second set was designed to test the sensitivity of the sensor to the flow rates (1–5 mL/s) of blood.

### 2.4. IoMT Multi-Bed Monitoring System

**Figure 5:**
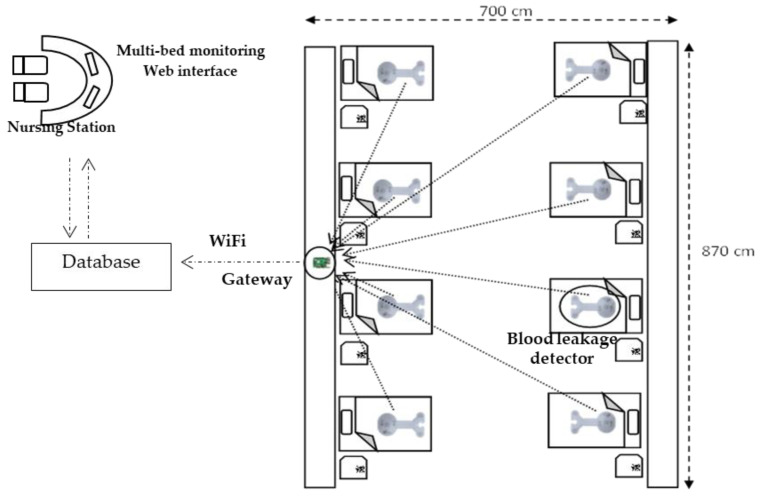
A diagram of the IoMT multi-bed monitoring system for leakage. The system could monitor needle dislodgement or blood leakage in real-time in multiple beds simultaneously.

In past studies, through the IoMT multi-bed monitoring system, the patient’s activity could be monitored, and the monitoring system could alert the nursing staff in time when a patient attempted to leave bed. The main results pertained to the number of falls, the time to turn off the out-of-bed alarm, and the number of out-of-bed attempts [12]. Our design, based on the architecture of the Acusense IoMT platform [13], was a real-time multi-bed monitoring system with at least one leakage-detection device, at least one IoT gateway, and a cloud analysis platform (Figure 5). A single gateway could accommodate for a maximum of eight blood leakage-detection devices. In the data collection, a multipoint Bluetooth 4.2 Nordic nRF52832 module was used, which was 2.4–2.48 GHz, 24 Mbit/s, −20 to +4 dBm and with a maximum transmission distance of 50 m. The information gathered by the IoT gateway was uploaded to the database through Wi-Fi. The role of the cloud analysis system was to save data packets for a long period (e.g., several years). When the IoT gateway system was connected to a wearable blood leakage-detection device, and the status of the monitoring system was normal with no alarm, a data packet conveying ‘normal’ was sent every 5 s. When the sensor patch detected any event of blood leakage, the alarm system gave a warning and a packet conveying ‘abnormal’ was transmitted once per second to ensure successful transmission to the cloud. In addition, the warning was displayed on the monitoring system to prompt the nursing staff to take immediate action.

### 2.5. Sensor Validation in the Clinical Trial

A total of 100 patients under hemodialysis were recruited as candidate subjects from Tainan Municipal An-Nan Hospital in Tainan City, Taiwan. First, we evaluated the clinical conditions of the participants. Their risk of leakage was scored using the blood-leakage risk questionnaire shown in Appendix A, Table A1. Participants without any risk factors or blood-leakage history in the previous 2 months were excluded, because the purpose of this trial was to test the performance of our system in detecting blood leakage. For this trial, we conducted two training sessions with 20 nurses, directing them on how to use our leakage-detection system and tested the system in five different areas of the hospital. The trial spanned over 2 months and was conducted in five shift periods. In total, 701 sessions of hemodialysis were monitored and recorded. The study design was approved by the Institutional Review Board of Tainan Municipal An-Nan Hospital (IRB number TMANH108-REC001). All subjects were informed of the objective and the procedure of this study, and all of them signed a written consent form.

**Figure 6:**
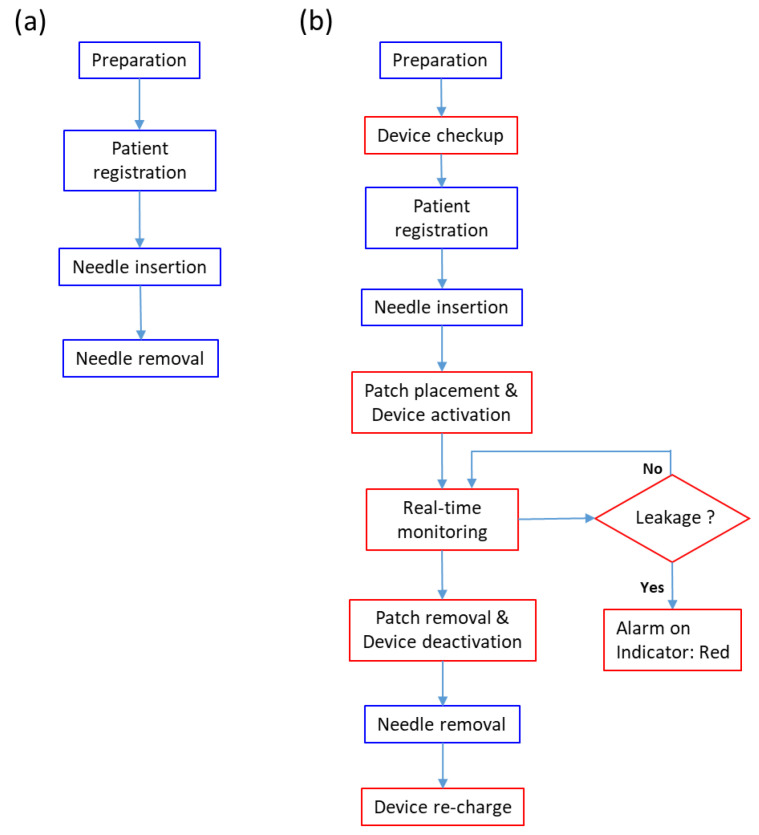
The operation steps (**a**) in a traditional hemodialysis session, and (**b**) in a hemodialysis session using our proposed leakage detection device. Blue boxes: standard hemodialysis steps. Red box: additional steps needed to use our leakage detection device.

The design of our system reflected the whole process of blood dialysis for hospitalized patients (Figure 6). To improve the adaptability in the clinical trials, we discussed the steps of hemodialysis with nurses and physicians. Tasks in blue boxes in Figure 6a are the typical operating procedures [14]. The steps included: (1) preparation before hemodialysis; (2) patient registration; (3) inserting the needle and performing dialysis; and (4) removing the needle and terminating dialysis. The red boxes in Figure 6b are additional steps for using our leakage-detection device. (1) The nurse should confirm the functionality of the leakage-detection device when preparing the device and supplies for dialysis. (2) After the nurse had inserted the needle into the hand of the patient, the patch should be connected to the warning device. The nurse should then turn on the warning device and attach it to the leading edge of the needle using medical tape. The nurse should then login to the monitoring system and configure the device. (3) The caregiver should confirm that the green light indicating normal status was on before dialysis began. (4) If blood leaked at this point, the buzzer would make a sound, and the light would turn red. The nurse should respond to the situation and record the leakage event. (5) After dialysis is completed, the nurse should discard the patch and clean the warning device with alcohol. The nurses should then logout from the monitoring system. (6) Finally, the nurses should charge the device for next use. In this study, the nursing staff received one month of training and then performed the pre-test before the monitoring system was implemented, and the post-test after the study. The nurses were then asked to complete a questionnaire with a researcher, which was scored using a 5-point Likert scale and collected within a week (Appendix A, Table A2). STATA software was used for all analyses.

The classification performance indicators of the blood leakage-detection device included accuracy, sensitivity, precision, specificity, F1-score and negative predicted value. F1-score was calculated as [15]

$$F1\text{-score}=2\times \frac{\mathrm{precision}\;\times \;\mathrm{recall}}{\mathrm{precision}+\mathrm{recall}}$$

## 3. Results

### 3.1. Pre-Clinical Simulation Experiment of Leakage Detection Patch

**Figure 7:**
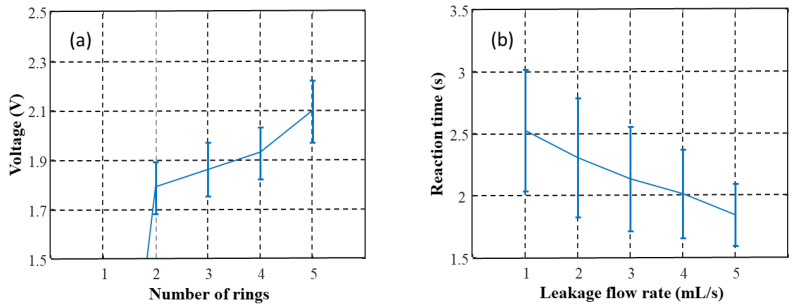
The relationship between the volume of blood leakage and (**a**) the sensor voltage and (**b**) the reaction time of the leakage detection patch, respectively.

**Figure 8:**
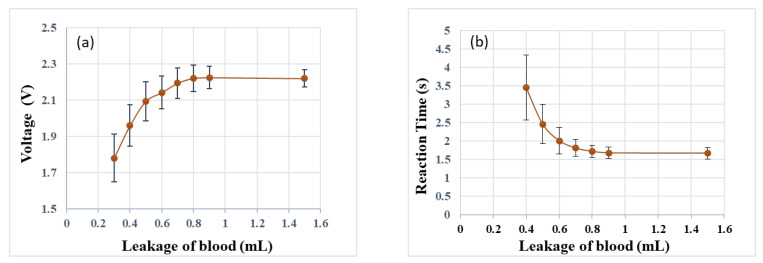
The relationship between the volume of blood leakage and (**a**) the sensor voltage and (**b**) the reaction time of the leakage-detection patch, respectively.

First of all, the sensor responses when the ejected fluid touched different numbers of sensor rings were measured. Each condition was repeated 10 times. The results (Figure 7a) showed that the sensor started to respond when two rings were touched by the fluid at the same time. The measured voltage increased as the fluid expanded to touch more rings. Then, the effects of the injection rate were tested. The results showed that the reaction time of the sensor decreased as the ejection rate increased (Figure 7b). In the third test, the effects of the ejected volume were studied. Each volume was repeated 10 times. The response of the leakage sensor increased non-linearly with the injected blood volume from 0.3 mL to around 0.8 mL and then reached a plateau (Figure 8a). There was a tendency for a larger variance with a smaller blood volume. The delay time between the injection of blood and triggering the alarm is shown in Figure 8b. The reaction time decreased with the volume of injected blood and became stationary at around 0.8 mL. Again, the variance decreased with an increase in the volume of blood. When the detection threshold was set as greater than 2 V, i.e., the blood leakage was greater than 0.5 mL blood leakage, the results revealed a detection rate of 100%.

In the last part, water (0.5 mL) was ejected in five different directions as shown in Figure 3b to test whether the sensor was equally sensitive to all directions. The ejection was repeated 10 times. The results showed that the measured voltages were 2.12 ± 0.09, 2.17 ± 0.10, 2.13 ± 0.10, 2.16 ± 0.10 and 2.14 ± 0.09 V for the directions A, B, C, D and E, respectively.

### 3.2. Test of the IoMT System with Multiple Users

With a network configuration as shown in Figure 5, a 30 min test was conducted to calculate the number of data packets received by the database. The test included normal-condition packets, the integrity of the packets, packets with unattached patches, leakage-detection packets, and historical leakage data. As mentioned in the Section 2, normal-condition packets and abnormal-condition packets were transmitted once per 5 s and 1 s, respectively. The overall success rate of the tests reached 100% with no lost data packets.

### 3.3. Sensor Validation in Clinical Trial

Fifty-two subjects were recruited and 701 sessions of hemodialysis were recorded. The characteristics of the enrolled hemodialysis patients are listed in Table 1. Twenty-two events of venous needle dislodgement (VND) or bleeding during dialysis were noted by the care faculty, of which 20 were correctly detected by our device. The confusion matrix is presented in Table 2. Of note, there were no false alarms in the 679 true negatives. No adverse reactions were reported by the patients. In summary, this clinical test achieved an accuracy of 99.7%, sensitivity of 90.9%, precision of 100%, specificity of 100%, F1-score of 99.9% and negative predicted value of 99.7%. These findings indicated that the proposed blood leakage-detection device was effective in detecting blood leakage.

### 3.4. Sensor Validation in the Clinical Trial

Twenty-two valid questionnaires were collected from the nursing staff in the pre-test period and 21 valid questionnaires were collected in the post-test period. The reliability of the questionnaires in all three dimensions were >0.7, including system experience (0.853), system operation (0.711) and nurse-patient interaction (0.711). In a comparison between the pre-test and the post-test periods, in terms of the impact of the use of the device, the general nursing staff in the pre-test worried that the use of the device would increase the burden and affect interaction. The values of burden and affect interaction in the pre-test period were 2.045 ± 0.213 and 2.227 ± 0.197, respectively. In the post-test period, the scores of these two aspects increased significantly to 2.857 ± 0.232 and 3.333 ± 0.311, respectively (Table 3). The differences in the burden and affect interaction scores between the pre-test and post-test periods were significant (0.014 and 0.005, respectively). The results indicate that the concerns of the nurses decreased after receiving training and using the device.

## 4. Discussion and Conclusions

**Figure 9:**
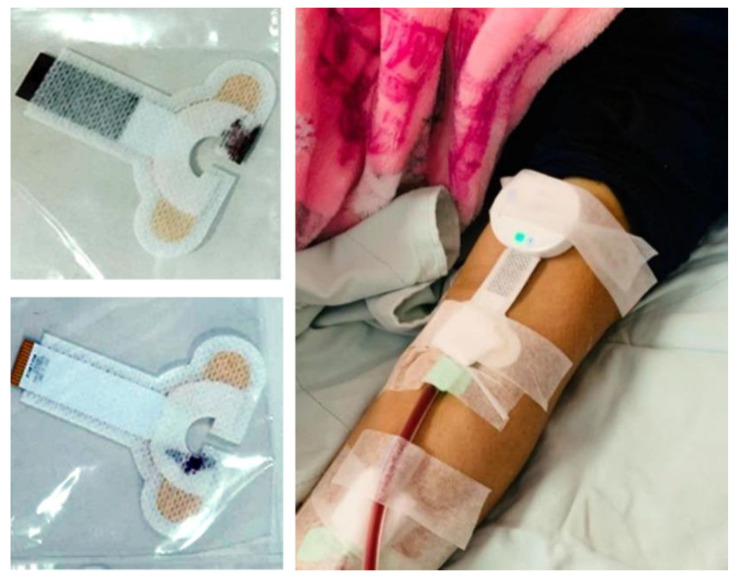
An example of the micro-leakage chart in the clinical trial. A tiny amount of blood infiltrated into a small area emphasized in a direction. The multi-ring design is better than a multiple point-type design for the estimation of leaked blood volume.

We previously designed multiple point-type patches [11], and found that the risk of blood leakage was positively correlated with the number of sensing points. Due to individual differences in the curvature of the arm surface, blood tends to leak in just one direction in clinical practice, and thus an array sensor with fixed sensor locations may underestimate the volume of leakage (Figure 9). In our design, each ring was connected to a resistor. When a small amount of blood passed through a ring by vampire cotton, it shortened the path length of the circuit, thereby reducing impedance and increasing the measured voltage. The distance between each ring and the needle differed, and thus, different voltage values were obtained for different volumes of blood leakage. Blood-leakage warnings could then be classified using a microcontroller that measured the voltage.

The results of the simulation tests revealed that different volumes of blood leakage corresponded to different voltage values. The results could be calibrated for blood-leakage volumes from 0.3 to 0.9 mL before the measurement saturated. This served as the basis to determine the amount of blood leakage and to set the threshold at which a warning would be triggered. The blood-flow rate of the access of hemodialysis has been estimated to exceed 500 mL/min [16]. Thus, the response time of a device used to detect blood leakage is a critical parameter. The response time of our proposed device was between 1.5 and 4.3 s, with a longer response time for milder leakage. The response time for blood leakage up to 0.8 mL was below 2 s. A previous study presented a blood-leakage detection device with a maximal response time of 3 s [11]. They reduced the radius of the inner circle of the patch in order to reduce the distance between the needle and the ring of the patch.

The human arm has a curved surface, and the curvature differs between individuals. Our sensor patch was designed to be attached to the arm. When a small amount of blood leaked persistently, it was less likely to spread out evenly in all directions, but rather to flow in one direction. Our results showed that the leakage mostly occurred on one side of the patch, although the direction was unpredictable. In other words, a circular configuration was necessary. The proposed cross-ring design can be customized in the future. For example, the distance between the second and first ring can be varied to balance between the response time and the amount of leakage. In addition, the distance between the inner ring and the needle can be varied to adjust the response time of the device. We can even change the blood-infiltration speed over the area by using different vampire cotton materials.

Clinically, our cross-ring design allows for customization of the threshold, and minimizes false alarms due to micro-leakages. In our previous study [11], the device could detect 0.1 mL blood and sound an alarm. However, it is unnecessary to sound an alarm for such a small amount of blood leakage. Therefore, the new design reduced the unnecessary workload of the nursing staff and provided a more accurate warning of blood leakage. Such a cross-ring sensing design can improve cost-effectiveness to create higher care quality for hemodialysis. In the present study, we also implemented a multi-bed-monitoring leakage detection system. The Acusense IoMT platform, a wireless IoT communication technology, was used as the hemodialysis unit. The results of software stability and function tests revealed that this wireless communication technology could accurately and reliably transmit information packets.

Through IoMT technology, nurses can be immediately notified of any blood leakage and the patients can be monitored in real time. In practice, quilts are commonly used to cover the arms of many patients. The nursing staff must therefore frequently move the quilt to inspect the needle puncture site. Previous devices have only had alarms on the device, and the sound was fairly quiet, especially when the arm was covered with a quilt. Using the Acusense IoMT platform, a message can be sent to the nursing staff from the monitoring system in real time. The results of the questionnaires revealed that this change increased patient safety and also increased the quality of patient care without increasing the operational burden of the nursing staff. However, a temporary disconnection could occur when a nursing staff member or a nursing work-cart blocks the Bluetooth transmission signal. In the future, the gateway devices can be placed on the ceiling to avoid them being blocked by objects.

The process of pairing and un-pairing a bed with our device is relatively time-consuming. The time is mainly spent on confirming and operating the interface of the monitoring system. One solution is to complete the pairing in advance, and the nurses can then simply use switches to control which and how many beds are in use, making the process much easier and less time-consuming. Another Bluetooth-related problem is that all nurses receive all warning signals without consideration of their assigned responsibilities. The system should therefore be modified so that only the hemodialysis beds for which the nurses are responsible appear on their monitors.

This study evaluated the performance of our leakage-detection system. The results obtained after conducting 701 sessions of hemodialysis, which is more than the number for a previous study of 544 sessions, demonstrated that the system provided 99.7% accuracy, 90.9% sensitivity, and 100% precision. The accuracy is relatively higher than that reported in a previous study (98.9%), mainly because of the additional IoMT system and patch design [12]. Two of the 22 leakage events were not detected by the system. These errors occurred because the blood did not penetrate the cotton layer of the patch, as there is an impermeable layer of nonwoven fabric in the patch where the blood can get stuck. This may also be the reason why the gauze on top of the needle changed the flow direction of the blood or the offset between the needle and the patch. The area of the nonwoven fabric of the patch should be reduced in future modifications.

With regard to extended application, this system can also be applied to home hemodialysis. The IoMT system can use a gateway in the home environment and upload information to the cloud, allowing centralized medical staff to monitor the dialysis process in a home environment. There are many similar devices for blood-leakage detection in the market. However, these devices are stand-alone products with no reliable multi-user, remote alarm system. The Acusense IoMT monitoring system can not only be implemented in dialysis centers, but also be applied to the home environment [17]. During the COVID-19 pandemic, it is even more important to implement a home telemonitoring system to improve the quality of in-home dialysis and avoid the risks of VND [18,19]. Our novel detector combined with an IoMT system is an effective automatic multi-bed monitoring system for blood leakage. It could more accurately control the threshold of different blood leakages, avoid unnecessary micro-leakage warnings, reduce the cost of care and achieve higher economic benefits. The clinical study using the system showed good performance with no major side effects.

## Figures and Tables

**Table 1:** Characteristics of the enrolled hemodialysis patients.

Variable	Percentage
Age (years)	62.8 ± 1.3
Male	69.2%
High risk of blood leakage	88.5%
The fistula had expired in the past month	40.4%
Diabetes	65.4%
Hypertension	67.3%
Sleeping pills	21.2%
Intradialytic hypotension	26.9%
Intradialytic hypertension	23.1%
Oral warfarin exposure	21.2%
Intradialytic anticoagulant (Units)	1138 ± 106

**Table 2:** Confusion matrix.

		True Condition	
		Positive	Negative
Predicted condition	Positive	20	0
	Negative	2	679

**Table 3:** The results of the questionnaires from the nursing staff.

Topic	Pre-Test ^#1^	Post-Test ^#1^	*p*-Value
I feel that detecting system can make me feel trust.	4.00 ± 0.15	3.76 ± 0.18	0.314
I feel that the detection system can make me feel at ease.	3.68 ± 0.17	3.76 ± 0.21	0.764
I think detecting system can help me reduce stress.	3.77 ± 0.15	3.76 ± 0.22	0.967
I am looking forward to using the detection system in care.	4.36 ± 0.16	4.43 ± 0.15	0.763
I feel that the detection system affects my interaction with patients.	2.05 ± 0.21	2.86 ± 0.23	0.014 *
I think detecting products will increase the workload of the operation.	2.23 ± 0.20	3.33 ± 0.31	0.005 *
I think the detection device is difficult to install.	1.86 ± 0.17	2.19 ± 0.18	0.185
I am able to provide the patients receiving hemodialysis with comprehensive care.	4.50 ± 0.14	4.38 ± 0.15	0.564
I think I can take the initiative to care about the patient during dialysis.	4.00 ± 0.20	3.91 ± 0.24	0.760
I think the patient is satisfied with the interaction during dialysis.	3.68 ± 0.26	2.86 ± 0.28	0.036 *

^#1^ Values in the form of mean ± one standard deviation. * Statistically significant difference between Pre-Test and Post-Test groups.

## Data Availability

The data that support the findings will be available in An Nan Hospital following an embargo from the date of publication to allow for commercialization of research findings.

## Appendix A

**Table A1:** Blood leakage risk questionnaire (Yes = 1 and No = 0).

1	The patient has frequent dialysis hypotension or spasms.
2	The patient is unconscious, uncontrollable, or unable to express words clearly.
3	It is difficult to access the needle or the injection angle or position needs to be adjusted.
4	In the past 3 months, the patient had blood leakage.
5	The injection site on the patient is not easy to fix or cannot be properly fixed.
6	The patient is allergic to the standard tape used for fixation.
7	The nurse cannot see the needle position. (for example: covered with a quilt or blanket)
8	The patient must often suspend dialysis and go to the toilet.

**Table A2:** The questionnaire for the nursing staff.

Code	Topic	Answer “No”
**ND1**	I feel that detecting system can make me feel trust.	0
**ND2**	I feel that detecting system can make me feel at ease.	0
**ND3**	I think detecting system can help me reduce stress.	0
**ND4**	I am looking forward to using the detection system in care.	0
**NX1**	I feel that detecting system affects my interaction with patients.	1
**NX2**	I think detecting products will increase the workload of the operation.	1
**NX3**	I think the detection device is difficult to install.	1
**NI1**	I am able to provide the patients receiving hemodialysis with comprehensive care.	0
**NI2**	I think I can take the initiative to care about the patient during dialysis.	0
**NI3**	I think the patient is satisfied with the interaction with the patient during dialysis.	0

ND: System experience, NX: system operation and NI: Nurse-patient interaction.
